# Supplementary material for: A Global Screen for Assembly State Changes of the Mitotic Proteome by SEC-SWATH-MS
Source: Cell Syst. 2020 Feb 26;10(2):133–155.e6. doi: 10.1016/j.cels.2020.01.001 (PMC7042714; doi:10.1016/j.cels.2020.01.001)

P11940 | PABP1_HUMAN | PABPC1 PAB1 PABP1 PABPC2
Monomer MW [kDa]: 70.671 Monomer expected elution fraction: 43

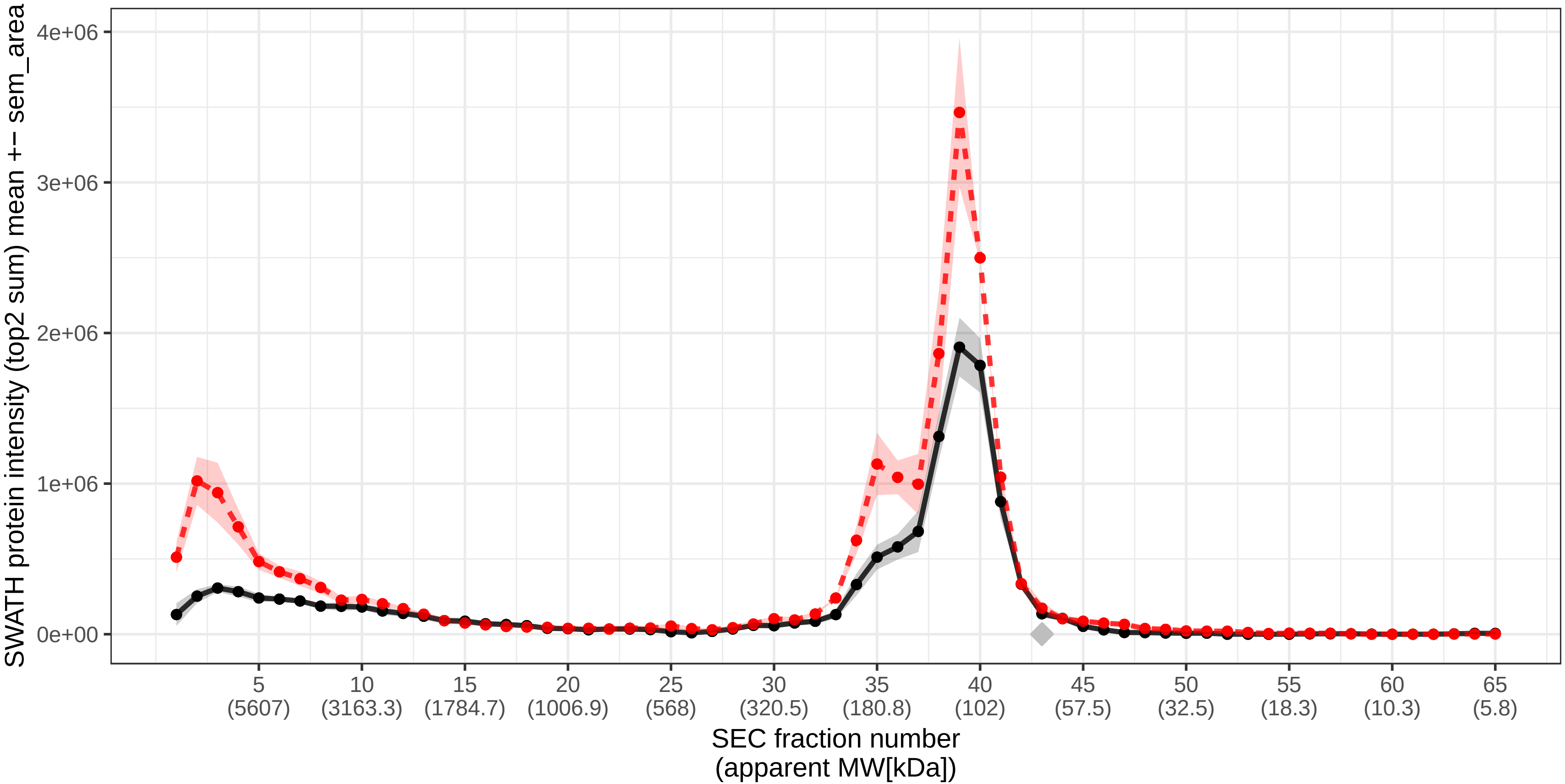

Supplement: Data S1. SEC-SWATH-MS Protein Chromatograms, Related to Figure 1 [file mmc6.zip › SECchrom_P11940_PABP1_HUMAN_PABPC1_PAB1_PABP1_PABPC2.pdf]
